# Juvenile Canine Leishmaniosis: A Systematic Literature Review and an Atypical Clinical Case

**DOI:** 10.3390/vetsci12070653

**Published:** 2025-07-10

**Authors:** Rosanna Dizonno, Oana Gusatoaia, Annamaria Uva, Floriana Gernone, Riccardo Paolo Lia, Andrea Zatelli, Maria Alfonsa Cavalera

**Affiliations:** 1 Istituto Zooprofilattico Sperimentale della Puglia e Basilicata, 71121 Foggia, Italy; 2 Department of Veterinary Medicine, University of Bari, 70010 Valenzano, Italy

**Keywords:** dermatological lesions, *Leishmania infantum*, lymphadenomegaly, papulo-nodular dermatitis, puppy, vertical transmission

## Abstract

Canine leishmaniosis (CanL), caused by *Leishmania infantum*, is a major zoonotic disease primarily transmitted by sand flies. Unlike in adult dogs, the clinical course of CanL in puppies remains poorly characterized, regardless of the transmission pathway (i.e., vertical transmission or vector exposure). This study presents the first systematic literature review (SLR) focused on juvenile CanL, alongside an atypical clinical case report. A PRISMA-compliant search across four databases identified three eligible studies describing CanL in puppies (≤9 months, according to the current canine life stage guidelines). The case involves a 4.5-month-old puppy adopted from southern Italy with papulo-nodular skin lesions and generalized lymphadenomegaly as well as a mild normocytic normochromic anemia and increased C-reactive protein. *L. infantum* infection was confirmed by serology, polymerase chain reaction (PCR), and cytology. The SLR suggests that dermatological lesions and/or lymphadenomegaly, whether associated with laboratory abnormalities, represent the most common clinical manifestations of CanL in puppies. In the presented case, the coexistence of systemic dissemination signs and papulo-nodular skin lesions, typically associated with vector-borne transmission, suggests the possibility of a dual route of infection by *L. infantum*. Juvenile CanL should be considered in differential diagnoses and supported by thorough diagnostic evaluation and appropriate follow-up protocols.

## Simple Summary

This study presents the first systematic literature review (SLR) on juvenile canine leishmaniosis (CanL) by *Leishmania infantum*, along with an atypical clinical case report. A PRISMA-compliant search across four databases identified three eligible studies describing CanL in puppies (≤9 months). The case involves a 4.5-month-old puppy adopted from southern Italy with papulo-nodular skin lesions and generalized lymphadenomegaly as well as a mild normocytic normochromic anemia and increased C-reactive protein. The SLR suggests that dermatological lesions and/or lymphadenomegaly, whether associated with laboratory abnormalities, may represent frequent clinical manifestations of CanL in puppies. In the presented case, the coexistence of systemic dissemination signs and papulo-nodular skin lesions, typically associated with vector-borne transmission, may suggest the possibility of a dual route of infection by *L. infantum*. Juvenile CanL should be considered in differential diagnoses and managed with comprehensive diagnostic workup and follow-up.

## 1. Introduction

Canine leishmaniosis (CanL) is caused by *Leishmania infantum*, a species previously known as *Leishmania chagasi* in the New World, and represents a significant, potentially fatal zoonotic infection in Africa, Asia, the Americas, and Europe [1,2]. The domestic dog serves as the primary reservoir for human infection [3].

Among countries where the *Leishmania* spp. are endemic, Brazil represents one of the largest foci of human and canine visceral leishmaniases, with thousands of cases reported annually [4]. In the Mediterranean regions, infection prevalence can reach up to 60% in dogs living in endemic areas [3]. Additionally, an increasing trend in the number of dogs without a travel history to CanL-endemic countries has been observed in central and northern Europe [5]. Italy is historically recognized as an endemic country for CanL, with an average *L. infantum* seroprevalence of 18.6% in 2017 [6]. Over the period 2009–2019, prevalence rates of 21.6% in northern Italy, 29.6% in central Italy, and 28.2% in southern Italy and the islands have been reported as well [7].

As a vector-borne pathogen (VBP), the protozoan *Leishmania* spp. is transmitted by the hematophagous activities of female phlebotomine sand flies belonging to the genera *Lutzomyia* (New World) and *Phlebotomus* (Old World) [1].

Though the sand flies bite is undoubtedly the primary route of *Leishmania* spp. transmission, vertical transmission should not be underestimated, particularly in puppies too young to have been exposed to vectors [8,9]. Sexual and transplacental transmissions of this protozoan have been documented in mice [10], humans [11,12], and dogs [13,14,15]. The parasite has been detected in biological samples from stillborn or newborn puppies [16,17] and symptomatic or asymptomatic naturally infected bitches [18]. Additionally, *Leishmania* infection has been associated with necrotizing placentates, abortion, and macroscopic or microscopic changes in the placenta [19]. Collectively, these findings provide evidence supporting the vertical transmission of *Leishmania* spp.

In adult dogs, clinical manifestations of CanL cover a broad spectrum, from asymptomatic infection to overt, multisystemic disease, depending on the prevalent host immune response type [20]. According to current guidelines [21,22], dogs may exhibit a plethora of clinical signs based on the degree of exposure, presence of parasitic infection, clinical presentation, and severity of organ involvement. Some dogs remain clinically healthy despite seropositivity or low-level parasite detection, while others develop mild-to-moderate clinical signs such as lymphadenomegaly or dermatological lesions, even in the absence of significant clinicopathological abnormalities. In more advanced stages, affected dogs may exhibit systemic signs including exfoliative dermatitis, weight loss, anemia, hyperglobulinemia, or proteinuria. In severe cases, the disease may progress to chronic kidney disease, vasculitis, ocular or joint complications, and ultimately end-stage renal failure or immune-mediated comorbidities [21,22].

Differing from adult dogs, the clinical course of CanL in puppies remains poorly characterized, regardless of the transmission pathway. The current staging systems are based primarily on observations in adult dogs and may not accurately reflect disease expression in puppies. Data on early-onset CanL are limited and fragmented, making it difficult to establish standard diagnostic or prognostic frameworks for this dog population [23,24]. Moreover, the immune response of puppies may differ substantially from that of adults, potentially influencing both susceptibility and clinical presentation [25]. Furthermore, juvenile CanL, particularly when left undiagnosed, may represent not only a veterinary health concern but also a potential public health issue, given the zoonotic potential of *L. infantum*. This gap in knowledge highlights the need for more focused investigation into the manifestations and progression of CanL in puppies [birth to cessation of rapid growth (6–9 months), varying with breed and size] [26]. Therefore, this study aims to deepen current knowledge on juvenile CanL by conducting a systematic literature review (SLR) and presenting a detailed case report of early-onset disease.

## 2. Materials and Methods

### 2.1. Systematic Literature Review: Search Strategy

On 18 June 2025, a SLR was conducted in accordance with the PRISMA (Preferred Reporting Items for Systematic Reviews and Meta-Analyses) guidelines [27]. Four online literature databases (PubMed, Scopus, WoS, and BASE) were screened to identify all publications on visceral leishmaniosis in puppies. The literature search was performed using Boolean Operators AND and OR as follows: [(leishmaniosis OR leishmaniasis OR leishmania) AND (canine OR dog) AND (juvenile OR offspring OR puppy OR young OR immature OR pediatric)].

### 2.2. Systematic Literature Review: Inclusion and Exclusion Criteria

To be considered, the articles were required to meet the following inclusion criteria: (i) should be observational studies such as cross-sectional, longitudinal, case series, or case reports; (ii) should have an accessible abstract; (iii) should include a puppy/puppies (aged ≤9 months [26]). No language restrictions were applied. For articles written in languages other than English, Italian, Spanish, or Portuguese, translations were performed using a free online multilingual translation tool (DeepL Translator, https://www.deepl.com accessed on 20 June 2025). This allowed for accurate understanding of the content and consistent application of the inclusion and exclusion criteria.

Studies were excluded if they met any of the following criteria: (i) reviews, (ii) non-original articles; (iii) editorials and experimental or quasi-experimental studies; (iv) studies related to human cases or non-canine species; (v) studies reporting co-infections in addition to leishmaniosis; (vi) reports regarding *Leishmania* spp. other than *L. infantum* and *L. chagasi*; (vii) studies involving dogs older than nine months [26]; (viii) studies enrolling animals whose age was not explicitly stated in the text; (ix) studies involving puppies with no clinical signs consistent with CanL and/or with a post mortem diagnosis; (x) articles without data on clinical presentation and diagnosis criteria (i.e., at least one of the following techniques: serology, cytology, histopathology, and polymerase chain reaction (PCR)—with the exclusion of PCR on tissues samples from mucocutaneous lesions—and (xi) articles considered “off-topic”, defined as not directly related to CanL.

### 2.3. Systematic Literature Review: Selection Process

The entire selection process was conducted in a blinded manner by two independent reviewers (R.D., O.G.), using the Rayyan application (Rayyan QCRI, https://rayyan.ai accessed on 18 June 2025), a web-based tool designed to facilitate screening and selection of studies for systematic reviews through blinding, tagging, and conflict resolution functionalities. Following the removal of duplicates from the databases, the titles and abstracts of each document were subjected to a review based on the pre-established inclusion and exclusion criteria. The selected publications were read in full by both reviewers to either confirm their eligibility and extract the data or to exclude them. Any disagreement was handled by discussion or involvement of a third reviewer (M.A.C.) until consensus was reached.

### 2.4. Systematic Literature Review: Data Extraction

Using a Microsoft Excel^®^ (Version 16.97, Microsoft Corporation, Redmond, WA, USA) spreadsheet, the following data was extracted and recorded from the included studies: title, first author name, year of publication, journal, type of article, type of study, study location, number and signalment of enrolled dogs, clinical presentations, diagnosis techniques, therapy, and follow-ups. The extracted data were compared across studies, and common patterns or discrepancies were identified and discussed.

### 2.5. Systematic Literature Review: Risk-of-Bias Assessment

The risk of bias for the included studies was assessed using the Joanna Briggs Institute (JBI) Critical Appraisal Checklist for Case Series [28]. Ten domains, including inclusion criteria, outcome measurement, case selection, and data reporting have been evaluated. Each domain was rated as “Yes”, “No”, or “Unclear” depending on the degree of methodological transparency. Two independent reviewers (M.A.C. and O.G.) assessed each study, and discrepancies were resolved through discussion.

## 3. Results

### 3.1. Case Presentation

**Figure 1:**
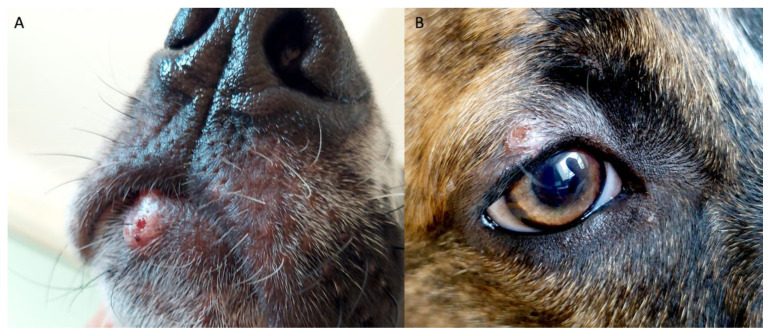
Dermatological lesions: (**A**) papulo-nodular, ulcerative dermatitis on the muzzle; (**B**) exfoliative dermatitis on the right eyelid.

In October 2022, a 4.5-month-old mixed-breed female dog adopted from Messina, Sicily, was presented for examination at the Borghesiana Veterinary Clinic in Rome, Italy, for dermatological lesions and anorexia. A thorough review of its medical history revealed irregular vaccination protocols, inconsistent antiparasitic prophylaxis, and a previous episode of parvovirosis at 2.5 months of age, with complete remission. On physical examination, the body condition score (BCS) and the muscle condition score (MCS) were 3/9 (i.e., 1–4 under ideal, 5 ideal, 6–9 over ideal) and 3/4 (i.e., 1—normal muscle mass and 4—severe muscle loss), respectively [29,30]. Mucous membranes, body temperature, and capillary refill time were within normal limits. Dermatological lesions were observed in multiple locations, including the head (pinna, eyelids, and snout) (Figure 1) and the distal extremities of the limbs. These lesions were characterized by a papulo-nodular appearance (Figure 1), with some presenting a crusty surface. Additionally, the dog presented generalized lymphadenomegaly, as palpable lymph nodes (i.e., prescapular, retromandibular, and popliteal) were notably enlarged. Following informed consent from the owners, a blood sample was collected to perform a complete blood count (CBC) (ProCyte Dx Hematology Analyzer, IDEXX Laboratories Inc., Westbrook, ME, USA), serum biochemical panel including acute phase proteins (i.e., C-reactive protein [CRP]) (Beckman Coulter, Clinical Chemistry Analyzer AU680), serum protein electrophoresis (Capillarys 2 Flex Piercing, Sebia Italia S.r.l., Florence, Italy), and indirect immunofluorescent antibody test (IFAT) for *L. infantum* IgG (MegaFLUO^®^ LEISH, MegaCor Diagnostik GmbH, Hörbranz, Austria), *Ehrlichia canis* (MegaFLUO^®^ EHRLICHIA canis, MegaCor Diagnostik GmbH, Hörbranz, Austria), as well as *Rickettsia conorii* IgG e IgM (MegaFLUO^®^ RICKETTSIA conorii, MegaCor Diagnostik GmbH, Hörbranz, Austria) antibodies detection. In addition, a cytological examination of the dermatological lesions was conducted to rule out diagnostic hypotheses, including cutaneous histiocytoma, dermatophyte keratosis, lichenoid keratosis, eosinophilic furunculosis, leishmaniosis, and other skin diseases such as viral plaque papillomatosis. Furthermore, a cytological examination of the enlarged lymph nodes was conducted to exclude neoplastic conditions (e.g., lymphoma) as well as leishmaniosis. Moreover, an abdominal ultrasound was performed to assess the abdominal lymph nodes, given lymphadenomegaly of the palpable lymph nodes.

**Figure 2:**
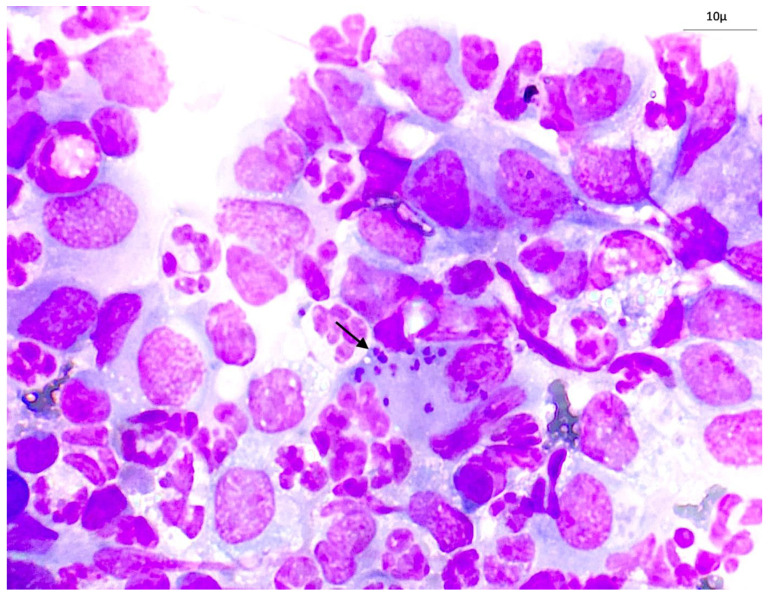
Fine-needle aspiration smear of retromandibular lymph node. Diff-Quik. 1000×. Macrophage engaged in the phagocytosis of *L. infantum* amastigotes (arrow).

At first examination, the CBC indicated a mild normocytic normochromic anemia [red blood cells (RBC) 4.56, reference interval (RI): 5.1–8.5 × 10^6^/μL; hematocrit (HCT) 30.7 RI: 36–56%)]. Biochemical results were within the reference ranges, except for an increase in phosphorus (8.04, RI: 2.3–5.5 mg/dL) and CRP (1.07, RI: 0.01–0.35 mg/dL) levels. The patient was seronegative for *E. canis* and *R. conorii* but seropositive for *L. infantum* by IFAT, with an antibody titer of 1:640. Cytology of the lymph node smear revealed the presence of *L. infantum* amastigotes phagocytosed by macrophages (Figure 2). In addition, a polymorphous lymphoid population was observed, along with a predominance of small and medium lymphocytes, as well as the presence of mature plasma cells and a considerable number of neutrophils. The cytological findings were consistent with mixed lymphadenitis resulting from the *Leishmania* spp. infection. Finally, direct observation of the parasite was also demonstrated in cytological smears of skin lesions.

Considering the laboratory findings and the clinical presentation consistent with CanL, treatment with allopurinol at a dose of 10 mg/kg every 12 h was started for 6 months, together with meglumine antimoniate at a dose of 100 mg/kg once daily by subcutaneous injection for 30 days. In addition, the attending veterinarian prescribed prednisolone at a dose of 1 mg/kg orally every 24 h for 3 days, 0.5 mg/kg orally every 24 h for 2 days, and 0.25 mg/kg orally every 48 h for four administrations, and fluralaner at 25 mg/kg per os, every 12 weeks.

One week after the initial examination, the dog still presented anorexia, dermatological lesions, and generalized lymphadenomegaly. The CBC, serum biochemical panel, including urea, creatinine, and phosphorus, complete urinalysis, and abdominal ultrasound were performed. The CBC still revealed a mild normocytic normochromic anemia (RBC 4.97 RI: 5.1–8.5 × 10^6^/μL; HCT 33.4, RI: 36–56%) and monocytosis (2.53, RI: 0.14–1.9 × 10^3^/µL), while biochemical results showed elevated phosphorus level (7.53, RI: 2.3-5.5 mg/dL). Urine dipstick analysis (Combur 9 Test, Roche, Rotkreuz, Switzerland) yielded a positive result for protein, classified as ‘+’ (30 mg/dL). In addition, the urinalysis revealed active sediment with the presence of occasional erythrocytes [2–5 RBC/high power field (hpf)], numerous leukocytes [6–10 White Blood Cells (WBC)/hpf], and numerous bacilli. The abdominal ultrasound examination showed moderate splenomegaly.

At the one-month follow-up visit, on physical examination, the dog still presented generalized lymphadenomegaly while dermatological lesions were undergoing remission. The CBC, serum biochemical panel including CRP, and a complete urinalysis plus urinary protein-to-creatinine ratio (UPC) on a urine sample collected by cystocentesis were performed. The CBC and the biochemical panel still revealed normocytic normochromic anemia (RBC 4.36, RI: 5.1–8.5 × 10^6^/μL; HCT 30.3, RI: 36–56%) and elevated phosphorus level (7.71, RI: 2.3–5.5 mg/dL), respectively.

At the three-month follow-up visit, no evidence of the previous clinical signs was observed, and the dog was in a good state of health. Blood and urine (by spontaneous micturition) samples were collected to perform a serum biochemical panel including CRP, serum protein electrophoresis, IFAT for *L. infantum* IgG antibodies detection, and urinalysis. The CBC and biochemical results were within the reference ranges, except for an increase in phosphorus (6.38, RI: 2.3–5.5 mg/dL) and a mild increase in CRP (0.39, RI: 0.01–0.35 mg/dL) levels. Urinalysis showed the presence of proteins and numerous bacilli (6–10 per field at 40× magnification). The patient was seropositive for *L. infantum* by IFAT, with an antibody titer of 1:80.

In February 2024, one year after the last follow-up visit, physical examination was unremarkable. The patient tested seropositive for *L. infantum* with a very high positive antibody titer detected by Enzyme-Linked Immunosorbent Assay (ELISA) (i.e., LEISCAN Ecuphar Veterinaria SLU, Barcelona, Spain) (6.4; RI: Negative < 0.9, Equivocal 0.9–1.1, Low Positive 1.1–1.5, High Positive 1.5–3.0, Very High Positive > 3.0). The CBC and UPC were within the reference ranges.

Two months later, in April 2024, physical examination was still unremarkable, and the CBC results were within the reference ranges. On the serum biochemical panel, which included phosphorus, creatinine, and urea, the latter parameter was elevated at 55 mg/dL (RI: 16.0–45.0 mg/dL). The patient remained seropositive for *L. infantum* but with a lower antibody titer, classified as low positive by ELISA (1.2; RI: Negative < 0.9, Equivocal 0.9–1.1, Low Positive 1.1–1.5, High Positive 1.5–3.0, Very High Positive > 3.0).

### 3.2. Literature Search Results

**Figure 3:**
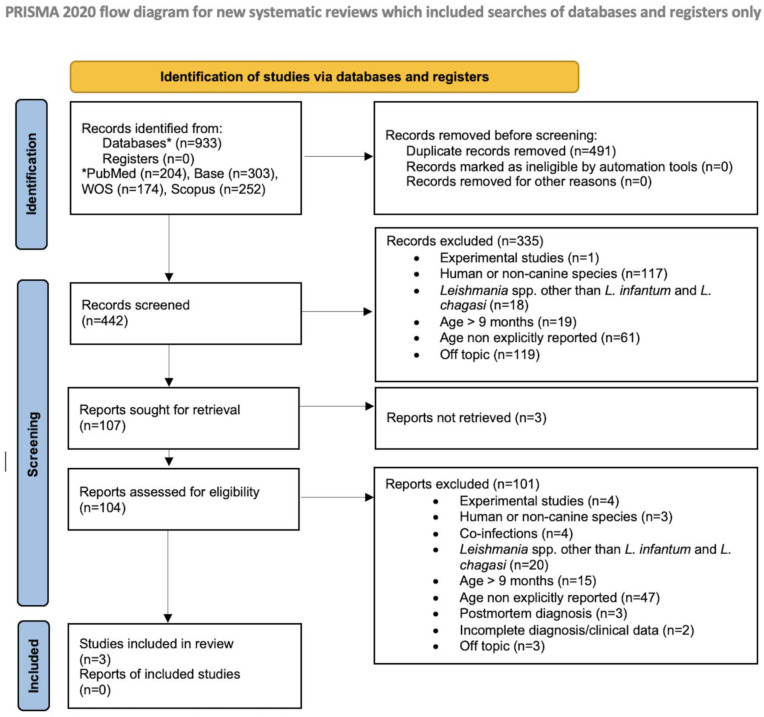
PRISMA 2020 flowchart illustrates the study selection process.

The workflow of the literature research based on the PRISMA guidelines for systematic reviews is shown in Figure 3. The total number of records identified from databases was 933. After the exclusion of 491 articles due to duplication, the remaining articles were screened by examination of the title and abstract. In addition, 488 articles were excluded during the screening process for various reasons. Among the records assessed for eligibility, one article was written in German and was therefore translated using the tool for translation. After full-text reading, it was subsequently excluded from SLR due to the enrollment of dogs older than nine months [31]. Finally, three studies were found eligible and included in this systematic review. The qualitative synthesis of the three studies (patient signalment, clinical signs, serological tests type and results, PCR/cytology results, laboratory abnormalities) is presented in Table 1.

**Figure 4:**
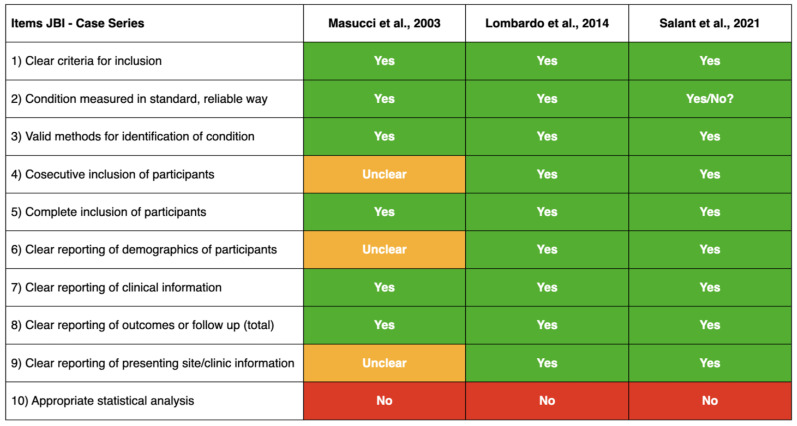
Risk of bias assessment according to the JBI Checklist for Case Series [28].

Included studies were appraised using the JBI Checklist for Case Series, as reported in Figure 4. Overall, the three articles fulfilled the majority of criteria, clearly defined inclusion criteria, and applied valid and standardized methods for condition identification and measurement, as well as clinical and outcome data. Consecutive inclusion of participants, clear reporting of demographics, and presenting site/clinic information were unclear in one study [18], whereas they were adequately addressed in the other two [32,33]. However, none of the studies reported statistical analysis.

In the study by Masucci et al. [18], 31 puppies born to seven seropositive bitches were evaluated. Although eight puppies (26%) tested PCR-positive on peripheral blood within the first month of life, only two of them exhibited clinical signs, specifically lymphadenomegaly, and were positive on lymph node samples both by PCR and cytology. These symptomatic puppies were born to a dam treated with N-methylglucamine antimoniate before pregnancy and did not receive therapy for CanL. A third puppy from the same dam initially tested negative at 17 days of age but later tested positive for *L. infantum* by PCR at 4 months of age, and no information on clinical signs was available. In addition, six asymptomatic puppies (19.3%) were born to another dam that had been treated with N-methylglucamine antimoniate both before and during pregnancy. At 26 days of age, five of these puppies (83.3%) tested PCR-negative, while one (16.7%) tested PCR-positive for *L. infantum*. One of the initially negative puppies tested positive when re-evaluated at 8 months of age. Overall, 6 out of 31 puppies (19.3%) which were PCR-negative at the initial examination (3 to 4 days of age) tested positive at follow-up (i.e., 45 days of age).

In the study by Lombardo et al. [32], 10 out of 17 dogs enrolled were herein considered due to their appropriate age (≤9 months). Three out of ten (30%) puppies were examined due to chronic non-pruritic cutaneous lesions (#6, #7, #8; Table 1) while the remaining seven were evaluated during routine check-ups. In two puppies (#6, #7; Table 1), cutaneous lesions had been present chronically from 3 weeks prior to presentation. In one case (#8; Table 1), lesions had been present from 1 month, while for the remaining puppies, the duration of the skin lesions was unknown. Detailed descriptions of the skin lesions of considered puppies are reported in Table 1. No additional abnormalities were found on physical examination in any of the puppies, except for one case (#6; Table 1), which exhibited mild solitary lymph adenomegaly of the regional popliteal lymph nodes. Cytological evaluation of regional lymph nodes was performed in six puppies. In puppies #1 and #6, cytology revealed reactive hyperplasia. In puppies #4 and #8, no abnormalities were found, while in puppies #2 and #7, cytology was non-diagnostic. Lymph node culture was negative in all puppies analyzed. Most puppies were seronegative by IFAT, except for puppy #3 and other two puppies (#9, #10) that presented low antibody titer of 1:80 and 1:320, respectively. All puppies were treated with N-methylglucamine antimoniate (100 mg/kg SC q24h) for 25–30 days. Puppies #2, #3, #4, #6, #7 were clinically evaluated 25 days after therapy but were subsequently lost to follow-up. In all puppies, after therapy, the cutaneous lesions had disappeared or evolved into a depigmented, flattened scar. Three dogs (#8, #9, #10) were also assessed for *Leishmania* infection between two and five months post-treatment. Puppy #8 remained seronegative, while the other two showed a decrease in antibody titer by IFAT (i.e., 1:40 and 1:80, respectively). Moreover, blood, lymph node, and conjunctival RT-PCRs were negative. Long term follow-up (6–24 months post-treatment) was obtained in four dogs (#1, #5, #9, #10). Among these, puppies #9 and #10 were assessed only by physical examination, which was unremarkable, while the other two (#1, #5) were also retested for *L. infantum* by IFAT with negative results and by RT-PCR performed on blood, lymph node, conjunctival, and oral swabs, also with negative results.

In the study by Salant et al. (2021) [33], seven out of nine (77.7%) puppies presented clinical signs potentially attributable to CanL (Table 1). Only one puppy out of seven (14.3%) presented also lymph adenomegaly, seropositivity for *L. infantum* by ELISA, and laboratory findings typically associated with CanL’s active form. Among the remaining six puppies, four dogs presented with dermatological lesions but showed no laboratory abnormalities, although they tested PCR-positive on various tissue samples. From a serological perspective, two symptomatic puppies were seronegative, despite testing positive for *L. infantum* by PCR (Table 1). All puppies exhibiting clinical signs were treated with allopurinol at a dosage of 10 mg/kg twice daily. Dermatological abnormalities improved within three weeks, and complete clinical recovery was achieved in all affected dogs within three months. After one year of treatment, all treated animals remained in clinical remission despite being seropositive for *L. infantum*, although their antibody titers had decreased.

## 4. Discussion

This study presents the first SLR on juvenile CanL with a total of three articles identified as eligible for a qualitative synthesis [18,32,33]. In addition, a case report describing a 4.5-month-old mixed-breed female dog, adopted from southern Italy, affected by an “unusual” clinical presentation of juvenile leishmaniosis has been presented.

The first article included in the qualitative synthesis following the SLR, by Masucci et al. [18], reported that 2 out of 31 puppies (6.4%) exhibited lymphadenopathy as the sole clinical sign consistent with CanL, testing positive for *L. infantum* by PCR on both peripheral blood and lymph node samples as well as by cytology on lymph node aspirates. Given that both puppies were only 17 days old at the time of the first examination, it is plausible that the early stage of infection precluded the development of further clinical signs. This hypothesis is supported by other studies indicating that, in natural infection, clinical manifestations of CanL may not appear until several months after exposure [34,35,36].

The second study included in the SLR, conducted by Lombardo et al. [32], reported that all dogs enrolled presented papular dermatitis. Interestingly, only 1 out of 10 puppies (10%), exhibited popliteal lymphadenopathy. This puppy was seronegative by IFAT, PCR-positive on conjunctival and oral swabs, and cytology-positive on skin lesions. The puppy was 4 months old at the time of diagnosis and, like the other dogs included in the study, originated from Catania and Palermo (Sicily), an endemic area for *L. infantum* in Italy. Only 3 out of 10 puppies (30%) were seropositive for *L. infantum* by IFAT, with low antibody titers (i.e., 1:80 and 1:320). These seropositive puppies were the oldest in the group (i.e., 7 and 8 months of age). Therefore, the *L. infantum* seronegativity and/or seropositivity observed may be age-related, considering that the median time for seroconversion under natural conditions is estimated to be approximately five months [24].

In accordance with Lombardo et al., (2014) [32] and by contrast with Masucci et al., (2003) [18], the third study included in the review, by Salant et al. (2021) [33], described 7 out of 9 puppies (77.8%) presenting with dermatological lesions compatible with CanL. The severity of clinical signs varied, with only one puppy exhibiting peripheral lymphadenopathy and laboratory abnormalities suggestive of CanL [24], while another displayed gastrointestinal signs (i.e., diarrhea and vomiting) along with conjunctivitis. These differences in clinical presentation compared with Masucci et al.’s study [18], may be related to the age of the dogs (ranging from 2 to 3.5 months of age, except for one that was 6 months old at the time of first presentation) and their breed (i.e., mixed Rottweiler), known for high susceptibility to developing clinical leishmaniosis [21,35,37]. The potential role of breed predisposition and young age in the development of clinical CanL is further supported by additional case reports of juvenile CanL, such as the case described by Santos and colleagues [38], which was not included in this systematic review due to lack of specific information regarding the Leishmania species involved. The authors reported a clinical case of a 8-month-old Boxer dog, a breed also known for its high susceptibility to CanL [21,35,39], presented with polyarthritis and laboratory abnormalities similar to those partially observed in Salant et al. [33], as well as in our case report (i.e., mild normocytic, normochromic anemia, hyperproteinemia with hypoalbuminemia and hyperglobulinemia). Cytological examination on peripheral blood, lymph node, and synovial fluid revealed the presence of *Leishmania* spp. amastigotes, and serological testing for *L. infantum* by IFAT demonstrated a serum antibody titer of 1:640, consistent with the result observed in our clinical case.

Nevertheless, the clinical presentation in our case is partially consistent with the description by Lombardo et al., (2014) [32] and differs from that reported in the other two reviewed studies and the related literature, since the puppy exhibited evident papulo-nodular skin lesions (Figure 1). In this context, it has been suggested that, as in humans, papular lesions due to *L. infantum* typically develop at the site of parasite inoculation and multiplication, following sand fly bites, which often occur in sparsely haired skin areas such as the bridge of the nose, lips, and eyelids, as observed in our patient (Figure 1) [40]. Furthermore, our patient, born in May and adopted in August 2022 from Sicily, an endemic area for *L. infantum*, presented with skin lesions approximately three months later, in October of the same year. This observation is in line with experimental data by Aslan et al. (2016) [41], who reported papular lesion development at multiple bite sites in Beagle dogs three months after experimental infection with *L. infantum*. Moreover, papular dermatitis has been predominantly described in young dogs [41,42], typically under one year of age [40]. Dogs with papular dermatitis usually do not exhibit other clinicopathological abnormalities, and anti-*Leishmania* antibody titers are often negative or only weakly positive [43], as also reported by Lombardo et al. [32]. This dermatological presentation is generally associated with a robust, specific cell-mediated immune response and may resolve spontaneously within 3–5 months [32,42,43].

However, in contrast to the expected localized and self-limiting nature of papular dermatitis, the present case showed signs consistent with systemic parasite dissemination. This included generalized lymph adenomegaly with palpable lymph nodes (i.e., prescapular, retromandibular, and popliteal) notably enlarged, positive PCR results from peripheral blood and lymph nodes, high anti-*L. infantum* antibody titre by IFAT (i.e., 1:640), and positive cytology from lymph node aspirates (Table 1). In addition, laboratory abnormalities consistent with a CanL active form (i.e., mild normocytic normochromic anemia and increased CRP) were also present. Taken together, these findings suggests the hypothesis of systemic infection and raise the possibility of vertical transmission, given the young age of the puppy.

Thus, while vertical transmission is highly plausible in the puppies described by Masucci et al. [18] and Salant et al. [33], especially considering their very young age and environmental conditions unfavorable to vector activity, a different interpretation is warranted for the study by Lombardo et al. [32] and our case report. In the first case [32], the occurrence of papular lesions in young dogs, in the absence of other clinical signs and accompanied by either absent or low humoral responses but strong parasite-specific cellular immunity, was interpreted as a direct consequence of the first contact of an immunocompetent host and *Leishmania* parasites inoculated by sand flies in the skin [32]. In our case report, though vertical transmission may explain some of the clinical signs (e.g., generalized lymphadenomegaly) and laboratory abnormalities, the presence of papular dermatitis in areas typically exposed to sand fly bites also suggests possible vector-mediated transmission. Consequently, our case may raise the hypothesis of a dual route of infection of *L. infantum* (i.e., concurrent vertical and vectorial transmission), although this remains a speculative interpretation that will require further evidence to be confirmed.

It is important to note that the puppy described in the present case report had previously been affected by parvovirosis at 2.5 months of age. Therefore, it cannot be excluded that the virus-induced immunosuppression [44] may have contributed to the systemic dissemination of *L. infantum*. Given the relatively high prevalence of such viral infections in young dogs, this potential contributing factor should not be overlooked when evaluating the pathogenesis and clinical progression of juvenile CanL.

Beyond the scope of this study, the potential influence of the health status and anti-*L. infantum* treatments administered to the dams on the clinical outcomes of their litters requires consideration. Indeed, in the study by Masucci et al. (2003) [18], the 2 out of 31 puppies showing clinical signs (Table 1) were born to a dam that had been treated with N-methylglucamine antimoniate prior to pregnancy. The other six asymptomatic puppies described in the same study were born to another dam treated with the same compound both before and during gestation; five of these puppies were PCR-negative for *L. infantum* in lymph node samples and exhibited no clinical signs, while the sixth was PCR-positive and clinically healthy. In the study by Salant et al. (2021) [33], the dam of the affected puppies was clinically healthy but PCR-positive for *L. infantum* in both peripheral blood and lymph node samples and had not received any treatment prior to or during pregnancy. These findings suggest that treatment with antimonial compounds, known to reduce parasitic load and modulate cell-mediated immune responses [45], may positively influence the clinical outcome of leishmaniosis in puppies born to infected dams. This hypothesis is further supported by a case report described by Spada et al. (2011) [46], in which a 4-year-old female dog affected by leishmaniosis was treated with the same antimonial compound during pregnancy. The two surviving puppies were clinically, serologically, and molecularly monitored from birth up to one year of age, consistently testing negative for *L. infantum* infection. This observation supports the potential role of antimonial treatment during pregnancy in preventing clinical disease in the offspring [46]. Unfortunately, no information was available regarding the dams of the puppies described by Lombardo et al. [32] and in our case report.

Building on these observations regarding maternal treatment, it is also important to consider the therapeutic approaches adopted for the infected puppies described in this article. All puppies described in the study by Lombardo et al., (2014) [32] were treated with a non-standard protocol of one dose of N-methylglucamine antimoniate (100 mg/kg q24 h) for 25–30 days. Follow-up data demonstrated that cutaneous lesions resolved or evolved into flat depigmented scars after therapy in all puppies after 25 days of therapy. Short- and long-term monitoring revealed either negative or decreasing IFAT titers and negative RT-PCR results in most dogs, suggesting a favorable clinical and parasitological response to treatment. All clinically affected puppies described by Salant et al. (2021) [33] received oral allopurinol at a dosage of 10 mg/kg twice daily for one year. Improvement in dermatological signs was observed within three weeks, and complete clinical remission was achieved after three months of therapy. The dogs remained in remission throughout the treatment period. Conversely, the puppy described in our case report was treated for six months with the same dosage of allopurinol used by Salant et al. (2021) [33] and additionally received subcutaneous injections of N-methylglucamine antimoniate at a dosage of 100 mg/kg once daily for 30 days. One month after the initiation of therapy, dermatological lesions had resolved, although generalized lymphadenomegaly persisted. After three months of treatment, the IFAT antibody titer had markedly decreased to 1:80, and the puppy showed no further clinical signs until one year after when the patient experienced an increase in the *L. infantum* antibody titer by ELISA, in absence of clinical signs and laboratory abnormalities compatible with CanL [24]. According to current therapeutic guidelines for CanL [47], the first-line treatment protocol consists of a combination of meglumine antimoniate and allopurinol. Several studies have supported this approach, indicating that the number of treatment failures, defined as worsening physical condition, relapse, or death, was higher in dogs treated with allopurinol alone compared to those treated with meglumine antimoniate alone (82% vs. 45%) and higher in dogs treated with meglumine antimoniate alone than in those treated with the combination therapy (45% vs. 18%) [48]. Furthermore, combining allopurinol with meglumine antimoniate not only reduces the duration of antimonial therapy, making it more tolerable and cost-effective, but may also temporarily reduce the infectivity of positive dogs for sand fly vectors [49]. Although all clinically affected puppies described herein achieved disease remission through different treatment protocols, long-term follow-up studies on juvenile forms of leishmaniosis would be valuable. This is particularly relevant considering that, in our case, the puppy, despite having been treated with the recommended protocol [47], experienced an increase in the *L. infantum* antibody titer one year later, albeit without clinical signs.

This study has several limitations. First, the SLR included only three eligible studies. While this limited number precludes any definitive conclusions and restricts the possibility of statistical synthesis, it also highlights the current scarcity of comprehensive data regarding juvenile CanL. This gap in the literature underscores the need for further research specifically focused on this understudied population. Moreover, all three studies identified by the SLR lacked statistical analysis of the data, as highlighted by the risk of bias assessment. However, this limitation reflects the intrinsic nature of the included studies, which were observational case series rather than comparative or case–control designs, and therefore not intended to support statistical inference. Finally, the clinical case presented, although carefully documented over a long-term follow-up, is subject to the inherent limitations of anecdotal evidence. As such, the clinical and pathophysiological interpretations drawn from a single case must be considered speculative and should be viewed as a starting point for further investigation rather than conclusive evidence.

## 5. Conclusions

This systematic literature review and case report suggest that dermatological lesions and/or lymphadenomegaly, whether or not associated with laboratory abnormalities, may represent frequent clinical manifestations of CanL in puppies. Thus, leishmaniosis should be included in the differential diagnosis of compatible clinical presentations in puppies, and thorough diagnostic investigations, including serology, PCR, and cytology, are strongly recommended to ensure early detection and implementation of appropriate management strategies. Moreover, in the presented case, the coexistence of systemic dissemination signs and papulo-nodular skin lesions, typically associated with vector-borne transmission, may suggest the possibility of a dual route of infection by *L. infantum* in puppies. However, this remains a speculative interpretation based on a single clinical observation. Further studies with larger cohorts are warranted to explore the role of vertical or dual route transmission in the pathogenesis of juvenile leishmaniosis, as well as to assess the long-term efficacy of current therapeutic protocols in young dogs.

## Figures and Tables

**Table 1:** Data on gender, age, clinical signs, serological and molecular results for *L. infantum*, cytology findings, and laboratory abnormalities in puppies from the articles included in the systematic literature review [18,32,33] and our case report.

Author/Year	Puppy #/ Gender (M/F)/ Age (Months)	Clinical Signs	Serological Tests		PCR Positive Results (Tissue)	Cytology Positive Results (Tissue)	Laboratory Abnormalities
			IFAT (Pos/Neg)	ELISA (Pos/Neg)			
Masucci et al., 2003 [18]	#1/NA/17 ^#,^*	LAP	NA	NA	PB, LN	LN	NA
	#2/NA/17 ^#,^*	LAP	NA	NA	PB, LN	LN	NA
Lombardo et al., 2014 [32]	#1/M/6	Nose papules	Neg	NA	NA	SL	NA
	#2/M/6	Abdomen papules	Neg	NA	SL	SL	NA
	#3/M/8	Inner surface of the pinna papules	Pos 1:80	NA	CS, SL		NA
	#4/M/8	Inner surface of the pinna papules	Neg	NA	CS, SL		NA
	#5/M/3.5	Eyelid and abdomen papules	Neg	NA	SL		NA
	#6/F/4	Abdomen papules; popliteal LAP	Neg	NA	CS, OS	SL	NA
	#7/M/4	Abdomen papules	Neg	NA	CS, SL		NA
	#8/F/6	Inner surface of the pinna and nose papules	Neg	NA	SL	SL	NA
	#9/F/7	Abdomen papules	Pos 1:320	NA	SL	SL	NA
	#10/M/7	Abdomen papules	Pos 1:320	NA	SL	SL	NA
Salant et al., 2021 [33]	#1/F/2	Nasal, truncal and pinnal crusty ED; peripheral LAP; KCS; fever; right hind limb lameness	NA	Pos	NA	NA	Normochromic normocytic anemia; leukocytosis; hyperproteinemia; hypergammaglobulinemia; increased liver enzymes
	#2/F/2.5	Nasal alopecia; mild periocular ED	NA	Pos	LN, PB, CS	NA	No abnormalities
	#3/M/2.5	Pinnal and periocular ED	NA	Pos	PB, CS	NA	No abnormalities
	#4/F/2.5	Localized alopecia of nasal planum	NA	Neg	CS	NA	No abnormalities
	#5/M/2.5	Localized nasal ulcer; diarrhea and vomiting; conjunctivitis	NA	Pos	LN, CS	NA	Hypergammaglobulinemia
	#6/M/3.5	Pinnal alopecia	NA	Pos	LN, PB, CS	NA	Hyperproteinemia; hypergammaglobulinemia
	#7/M/6	Cutaneous nasal planum ulcer	NA	Neg	Skin	NA	No abnormalities
Case report	Puppy/F/4.5	Generalized LAP; papulo-nodular SL; anorexia	Pos 1:640	Pos	LN, PB, SL	LN, PB, SL	Mild normocytic normochromic anemia

Note: ^#^ puppies born from treated mother before pregnancy; * age in days. Abbreviations: CS, conjunctival swab; ED, exfoliative dermatitis; KCS, keratoconjunctivitis sicca; LAP, Lymphadenopathy; LN, lymph node; NA, not available; OS, oral swab; PB, peripheral blood; SL, skin lesions.

## Data Availability

Contained within this paper.
